# How can climate adaptation policy enhance urban climate resilience? Empirical evidence from China

**DOI:** 10.1371/journal.pone.0335736

**Published:** 2025-11-06

**Authors:** Ana Yin, Ziqi Liu

**Affiliations:** 1 Hebei Province Research Base for Science and Technology Innovation and Sustainable Regional Economic Development, Hebei GEO University, Shijiazhuang, Hebei, China; 2 School of Economics, Hebei GEO University, Shijiazhuang, Hebei, China; Yunnan University, CHINA

## Abstract

As the primary carriers of population aggregation, cities face severe climate risks and challenges. This paper examines whether and how climate adaptation policy can enhance urban climate resilience. Based on panel data from 57 cities in China’s typical ecologically fragile region—the Yellow River Basin—from 2010 to 2022, this study employs the Synthetic DID method to evaluate China’s Climate Adaptive City Construction Pilot Program (CACCP). The results indicate that the CACCP policy can significantly enhance urban climate resilience, and this conclusion remains valid after a series of robustness tests. The CACCP policy mechanism reveals that green bond instruments play an effective mediating role in promoting urban climate resilience under the CACCP policy. Urban education and public environmental concern have positive moderating effects on the implementation of the CACCP policy, while urban mineral resource dependency has an adverse moderating effect. These findings confirm the effectiveness of climate adaptation policies. It also reveals the financial tools, human capital, cultural values, and economic structural pathways that drive human adaptation to climate change.

## 1. Introduction

The IPCC Sixth Assessment Report (AR6) clearly states that human-induced climate change has already impacted numerous extreme weather and climate events worldwide, resulting in widespread adverse effects on food and water security, human health, and the economy and society [1]. Since the 21st century, although countries have made active efforts to mitigate climate change, current mitigation actions have failed to meet the targets of the Paris Agreement, and keeping global warming below 2°C has become increasingly challenging [1]. The international community has strongly urged all countries not only to strengthen climate change mitigation measures but also to accelerate climate adaptation efforts, which are now urgent and indispensable [2]. However, a gap remains between current adaptation levels and those required to address impacts and mitigate climate risks. Funding, knowledge, and practice gaps have been identified as significant systemic barriers [1].

Scholars have actively monitored the progress of climate adaptation policies and actions in various countries. Some scholars have employed textual analysis, questionnaire surveys, and case study methods to explore the planning and diffusion of climate adaptation policies [3,4], as well as the driving forces and obstacles [5,6]. A few scholars have evaluated the effectiveness of climate adaptation policies [7–9]. Fu et al. (2021) described the implementation process of the CACCP policy in China and constructed an index system for climate-adapted cities to evaluate the policy’s performance [7]. Jiang et al. (2023) used remote sensing data to analyze the impact of ecosystem restoration projects on mitigating land degradation [8]. Jee et al. (2024) developed a nonlinear flood damage function, used simulation and prediction techniques to assess the potential impact of a regional climate adaptation policy in Korea [9]. However, most assessment objectives of climate adaptation policies are constrained to quantifying outputs and delineating processes [10]. There is a need for more scientific evaluation of the impacts of climate adaptation policies and the mechanisms through which they operate.

In today’s world, cities are the primary hubs for population aggregation and economic activity. At the same time, the fragility of the urban ecological environments makes them more susceptible to the impacts of extreme weather shocks. In 2017, the National Development and Reform Commission and the Ministry of Housing and Urban-Rural Development in China jointly issued the Climate Adaptive City Construction Pilot Program (CACCP), establishing 28 pilot cities to improve urban climate adaptability. In 2022 and 2023, China successively issued the National Strategy for Adapting to Climate Change 2035 and the Notice on Deepening the Pilot Program for Climate-Adaptive City Construction, establishing a comprehensive national climate adaptation process. The practical effectiveness of China’s CACCP policy, particularly whether it can enhance climate resilience in typical ecologically fragile regions, is worthy of attention.

Since Holling, a Canadian ecologist, first introduced the concept of “resilience” into ecology [11], the idea of “urban resilience” has garnered widespread attention from managers and scholars. Scholars from different disciplines have defined “urban resilience” in various ways; however, most definitions lack clarity [12]. Meerow, Newell, and Stults (2016) proposed the 5W theoretical model of urban resilience, which comprehensively interprets urban resilience as encompassing the ability to recover, adapt, and transform [13]. In recent years, some scholars have argued that humans should examine the topic of urban resilience from a broader perspective, pointing out that an essential goal of urban resilience is for humans to adapt to change and thrive in life, thereby mitigating the impact of unexpected risks or pressures [14]. The quantitative methods of urban resilience primarily involve multi-dimensional indicator systems, including those designed for short-term disaster risk prevention [15,16] and integrated systems encompassing economic, social, ecological, and infrastructure aspects [17–19]. As climate issues are becoming a core risk and a massive threat to the sustainable development of cities, some scholars suggest planning urban climate adaptation strategies from the perspective of urban resilience [20].

In summary, relative to mitigation measures to address climate change, climate adaptation construction in most countries is still at an early stage of planning and implementation. Policy objectives primarily focus on short-term climate mitigation, and a lack of emphasis on climate adaptation will lead to more serious climate disaster losses. As climate issues increasingly become a core risk affecting sustainable urban development, some scholars have called on managers to plan urban climate adaptation strategies from the urban resilience perspective. Therefore, this paper harmonizes climate adaptation policy objectives with urban resilience and defines the concept of “urban climate resilience.” Then, it takes China’s CACCP policy as the research object and selects a typical ecologically fragile area—the Yellow River Basin urban agglomeration—as the research sample to explore how climate adaptation policy can enhance the urban climate resilience. The marginal contributions of this paper are mainly in the following three aspects. First, based on the perspective of human adaptation to climate change over the long term, this paper defines the concept of “urban climate resilience.” This research promotes the application and development of urban resilience theory, clarifying policy objectives for human adaptation to climate change. Second, this paper empirically analyzes the practical effects of China’s CACCP policy in typical ecologically fragile areas, revealing the effectiveness of climate adaptation policy planning and actions. This finding helps to strengthen the confidence of developing countries in climate adaptation actions. Third, this study further explores the mediating role of green bonds and the moderating roles of education, public environmental concern, and mineral resource dependency, revealing diversified driving mechanisms in climate adaptation policy governance.

## 2. Concept definition, policy background & research hypothesis

### 2.1. Concept definition

The “resilience” theory has undergone an evolutionary process, transitioning from “engineering resilience” to “ecological resilience” to “socio-ecological resilience.” Resilience can be traced back to engineering science and fluid mechanics [21], which describes the resistance of materials or structures to external shocks. In 1973, Canadian ecologist Holling first introduced the concept of “resilience” into ecology, arguing that ecological resilience measures the persistence of a system and its ability to absorb changes and disturbances [11]. Compared with the traditional ecological concept that emphasizes “steady state,” Ecological resilience emphasizes the dynamics of the system and multiple stable states. Based on the ecological resilience theory, Berkes, Folke, and Colding (1998) conceptualized nature and society as an interwoven system that develops together, i.e., the socio-ecological system [22]. They believe that socio-ecological resilience reflects the socio-ecological system’s persistence, adaptability, and transformability. These theories offer crucial insights into the complex socio-ecological systems and their sustainable management, particularly in addressing climate change and mitigating disasters.

Since the 21st century, global urbanization has brought unprecedented environmental and social challenges. As the social demand for reducing urban disasters increases, urban resilience has garnered the attention of managers and academics. Scholars from different disciplines have various definitions of urban resilience, but social theorists have sharply criticized these definitions’ lack of conceptual clarity. In response to critical comments, Meerow, Newell, and Stults (2016) proposed a new definition of urban resilience, which refers to “the ability of an urban system-and all its constituent socio-ecological and socio-technical networks across temporal and spatial scales-to maintain or rapidly return to desired functions in the face of a disturbance, to adapt to change, and to quickly transform systems that limit current or future adaptive capacity.” [13] At the same time, they proposed the 5W principle to interpret the connotation of urban resilience.

Unlike short-term disaster combating and mitigation planning in urban climate change response actions, climate-adaptive city construction should focus on urban resilience planning for long-term climate change [23]. The connotation and significance of climate adaptation should be broader than climate mitigation planning, which focuses on “carbon emission reduction” [14,20]. Especially in the current situation where global mitigation actions have failed to achieve the goals of the Paris Agreement and climate warming is increasing, only by focusing on improving the comprehensive capacity of cities to adapt to climate change in the long term can urban systems achieve high-quality, sustainable development. We define the concept of “urban climate resilience” as the comprehensive ability of cities to adapt to long-term climate change trends, encompassing stability, coordination, and transformation. Among these, “stability” reflects the ability of the city to quickly resist, self-recover, and stabilize the regular operation of the urban system when it is subjected to extreme climate shocks. “coordination” reflects the ability of the city to actively take measures to coordinate the relationship between economic growth and climate change. “transformation” reflects the ability of the city to promote the reshaping of its system structure and the transformation of organizational functions.

### 2.2. Policy background

China has attached equal importance to mitigation and adaptation in addressing climate change. While promoting the carbon reduction strategy, China actively explores constructing climate-adaptive cities. In February 2017, the National Development and Reform Commission and the Ministry of Housing and Urban-Rural Development jointly issued the CACCP policy, which, for the first time, established 28 cities (districts) as pilot areas for building climate-adaptive cities. In June 2022, China released the National Climate Change Adaptation Strategy 2035, outlining a comprehensive national strategy for climate adaptation. In August 2023, China issued a notice on deepening the CACCP policy. In May 2024, 39 cities (districts) were established as pilot cities for deepening the construction of climate-adaptive cities. Based on the above development of China’s climate adaptation policies, it is evident that the Chinese government attaches great importance to the severe trend of climate change. It promotes the combination of policy pilot experiments and a comprehensive approach, aiming to summarize the experience gained from practical exploration and seek progress.

### 2.3. Research hypothesis

China’s CACCP policy was issued and implemented in February 2017. The policy document clearly states that the objective is “to enhance cities’ ability to adapt to climate change.” It encompasses a range of strategies and actions aimed at reducing the vulnerability of urban systems to climate change and extreme weather events [24]. First, the CACCP policy has prompted local governments in pilot areas to actively engage in climate adaptation actions, including establishing special contingency plans, improving disaster prevention standards, and enhancing risk monitoring and early warning capabilities [25]. These actions will enhance cities’ ability to respond to sudden extreme climate events, improving their short-term resilience. Second, the CACCP policy was jointly issued by the National Development and Reform Commission and the Ministry of Housing and Urban-Rural Development. Its core is establishing a cross-departmental collaborative governance mechanism and a target coordination framework, integrating climate adaptation goals into mainstream urban planning, land use, and industrial policies. Through mandatory assessments, target breakdown evaluations, and fiscal incentives and penalties, the policy “forces” local governments to pursue green and low-carbon development paths [7]. This development path alleviates the contradiction between urban economic growth and carbon emissions reduction, directly enhancing urban climate resilience’s medium- and long-term coordination capacity. Third, the CACCP policy provides dedicated financial support to help pilot initiatives to overcome institutional barriers, such as establishing resilience demonstration zones and promoting climate-adaptive land use. The policy also encourages local authorities to introduce international best practices and technologies. It promotes the development of indigenous, climate-resilient technologies, including water recycling, passive energy-efficient buildings, and resilient agriculture [15]. These measures promote the forward-looking and fundamental restructuring of urban infrastructure, industrial structure, spatial layout, and management models, catalyzing and enhancing the long-term transformative capacity. Based on the above analysis, we put forward the following research hypothesis.

H1: The CACCP policy can directly enhance urban climate resilience.

The United Nations Sustainable Development Goals agenda significantly emphasizes the role of green investments [26]. SDGs 12 and 13 seek to stimulate investment in climate finance, thereby ensuring a low-carbon and climate-resilient global transition [27]. The role of financial markets in mitigating climate risks encompasses the development of financial innovations, such as green bonds, and a ramp-up in climate-aware mutual funds [28]. Scholars have posited that green bonds and climate funds can help reduce carbon emissions by investing in green projects for clean energy, pollution abatement, and infrastructure [29,30].

Since China formally established its green bond market in 2015, it has experienced rapid growth. In a 2022 interview, Sean Kidney, CEO of the Climate Bonds Initiative, stated, “In the field of green bonds, China started from scratch and developed into the world’s second-largest market in less than a decade.” China has incorporated the implementation and advancement of green bonds into its 14th Five-Year Plan and considers green finance a crucial instrument for attaining the objectives of “carbon peak” and “carbon neutrality” [31,32]. Green bonds have also been instrumental in international and domestic climate finance, particularly China’s development of carbon neutrality bonds in 2021, which have recently constituted over 45% of the green bond market. Some scholars have also presented theoretical analyses and empirical evidence suggesting that China’s green investment contributes to carbon emission reduction [33,34]. Based on the above analysis, we put forward the following research hypothesis.

H2: The CACCP policy enhances urban climate resilience by using green bonds as a financial innovation tool.

Human activities play a significant role in environmental issues such as anthropogenic climate change [35]. Education is a vital instrument for advancing human capital [36]. It delivers environmental curricula encompassing climate change mitigation, pollution abatement, and biodiversity conservation. Climate change curricula elucidate the mechanisms by which human activities impact Earth systems. Lessons on pollution and biodiversity loss expand comprehension of specific ecological disturbances [37]. Consequently, well-educated citizens demonstrate greater capacity to interpret and implement complex environmental policies [38]. Therefore, a higher level of education contributes to the better functioning of climate adaptation policies, and we propose the following research hypothesis.

H3: The CACCP policy effect can be optimized by attaining a higher level of education.

The theory of collective action postulates that the elevation of public consciousness can galvanize collective action and consolidate social power, thereby facilitating the implementation of environmental policies [39]. The theory of social capital posits that heightened public environmental concerns can enhance social capital and foster a social atmosphere of shared values and responsibility, which is conducive to promoting environmental policies [40]. Due to China’s economic growth, there has been a discernible surge in public environmental consciousness and involvement in environmental governance [41], which has proven instrumental in implementing environmental policies in China [42,43]. Consequently, we put forward the following research hypothesis.

H4: The CACCP policy effect can be enhanced by cultivating a greater sense of public environmental concern.

Mineral resources are indispensable for sustaining human life and production. Nevertheless, a high reliance on fossil fuels will amplify the carbon footprint of urban areas, leading to environmental contamination and ecological deterioration, which, in turn, will impede or even stall regional economic growth. As postulated by economists [44,45], the resource curse phenomenon represents a potential consequence of this situation. Therefore, high dependence on mineral resources for urban development is associated with heightened ecological pressure and vulnerability [46], rendering urban areas more susceptible to extreme climate events and impeding the advancement of climate adaptation strategies. Concurrently, a long-term economic development model that prioritizes the utilization of mineral resources will perpetuate the region’s reliance on a resource-based growth trajectory, hindering the implementation of strategies such as transitioning to low-carbon energy [47]. Therefore, from a reverse perspective, reducing dependence on mineral resources in urban development will facilitate the implementation of climate adaptation strategies. We propose the following research hypothesis.

H5: The CACCP policy effect can be optimized by reducing the dependence on mineral resources.

## 3. Methods and materials

### 3.1. Overview of the research area

The Yellow River is China’s second-longest river, spanning the country’s eastern, central, and western regions. Its total basin area is approximately 795,000 square kilometers. The Yellow River flows through nine provinces, including Qinghai, Sichuan, Gansu, Ningxia, Inner Mongolia, Shaanxi, Shanxi, Henan, and Shandong, covering 69 cities [48]. Among them, 36 are mineral resource cities, making the region a significant energy hub in China. However, the coal energy industry dominates the region’s economic development, which has led to the dual challenges of industrial transformation and carbon emission reduction. At the same time, the Yellow River Basin has a fragile ecological environment, characterized by frequent regional droughts and floods in recent years and a significant increase in extreme weather events [49]. The Yellow River Basin is identified as one of the key strategic regions in the National Climate Change Adaptation Strategy 2035. It proposes action measures such as “comprehensively implementing water conservation and control actions in the Yellow River Basin, strengthening ecological restoration and governance, promoting networked, integrated monitoring and assessment of climate change, fine-tuning the forecast, and constructing an integrated transportation network, infrastructure, and public service system.” This study assesses the potential of the CACCP policy to enhance urban climate resilience in the Yellow River Basin. Specifically, it provides insights pertinent to climate adaptation strategies in ecologically vulnerable regions. For the analysis, cities exhibiting substantial missing data from 2010 to 2022 were excluded. Consequently, the final sample comprises 57 cities, including nine pilot cities and 48 non-pilot cities.

### 3.2. Methods

#### 3.2.1. Policy effect model.

To examine the impact of CACCP policy on urban climate resilience in the Yellow River Basin, a model is constructed using panel data from 57 cities from 2010 to 2022, with 2017 as the year of policy implementation, as shown in Eq (1).


$$Resilience_{it}=\alpha_{0}+\beta treated_{it}+\sum_{j=1}^{3}\gamma_{j}X_{it}+u_{i}+\lambda_{t}+\varepsilon_{it}$$ (1)

Where i denotes city and tdenotes year. Resilience is the outcome variable representing urban climate resilience in this study. treated represents a dummy variable for policy treatment. When the city is in the experimental group, it is assigned a value of 1 after the policy is implemented and a value of 0 before it is implemented. When the city is in the control group, it is assigned a value of 0. Xrepresents the control variables. β is the estimated coefficient of the policy effect we are interested in, i.e., the average treatment effect. $\alpha_{0}$ is a constant term. $\gamma_{j}$ is the coefficient of the control variable. $u_{i}$ and $\lambda_{t}$ denote the city and year fixed effects, respectively. $\varepsilon_{it}$ is a random error term.

Given that the Synthetic DID method improves upon the traditional synthetic control method (SCM) and the difference-in-difference method (DID), its estimators exhibit better precision and robustness, particularly in scenarios involving small treatment group sizes [50]. In this study, with nine treatment groups and 48 control groups, we selected the Synthetic DID method for policy effect estimation. This method primarily consists of three steps: first, identify the individual weighting variable ${\hat{\omega }}_{i}^{sdid}$ such that the pre-treatment trend of the outcome variable in the control group is as close as possible to that in the treatment group; second, identify the time weighting variable ${\hat{\lambda }}_{t}^{sdid}$, which is used to weight the outcome variables prior to the event to match them with the outcome variables post-event; finally, incorporate ${\hat{\omega }}_{i}^{sdid}$ and ${\hat{\lambda }}_{t}^{sdid}$ into the DID two-way fixed effects model to estimate the average treatment effect, yielding the Synthetic DID estimate, as shown in Eq (2).


$$\left({\hat{\tau }}^{sdid},\hat{\mu },\hat{\alpha },\hat{\beta }\right)=\underset{\tau ,\mu ,\alpha ,\beta }{\mathrm{\backslash arg}\min}\left\{\sum_{i=1}^{N}\sum_{t=1}^{T}{\left(Y_{it}-\mu -\alpha_{i}-\beta_{t}-W_{it}\tau \right)}^{2}{\hat{\omega }}_{i}^{sdid}{\hat{\lambda }}_{t}^{sdid}\right\}$$ (2)

where ${\hat{\tau }}^{sdid}$ denote the average treatment effect estimates from the Synthetic DID method. N and t denote the units and time of the balanced panel data. Treatment variables are denoted as $W_{it}\in \left\{0,1\right\}$. $\alpha_{i}$ and $\beta_{t}$ denote individual fixed effects and time-fixed effects. μ is a random error term. ${\hat{\omega }}_{i}^{sdid}$ and ${\hat{\lambda }}_{t}^{sdid}$ denote individual weights and time weights, respectively.

#### 3.2.2. Mediating effect model.

To examine the mediating mechanism role of green bonds in the implementation of the CACCP policy, this paper constructs a mediating effect model based on the model and the Sobel test methods proposed by Wen et al. (2022) [51], as shown in Eqs (3) - (5). Eq (3) represents the total effect of the CACCP policy, while Eq (4) and (5) represent the CACCP policy's pathway to enhance urban climate resilience through the use of green bonds. In this model,GBdenotes green bonds and represents the total amount of municipal green bond issuance. The meanings of the other variables are consistent with those in the model (1). The stepwise regression method indicates the presence of a partial mediation effect if ${\hat{\beta }}_{1}$, ${\hat{\beta }}_{2}$, ${\hat{\beta }}_{3}$, and $\hat{\delta }$ are significant, and a full mediation effect if ${\hat{\beta }}_{3}$ is not significant and the others are all significant [52].


$$Resilience_{it}=\alpha_{0}+\beta_{1}treated_{it}+\sum_{j=1}^{3}\gamma_{j}X_{it}+u_{i}+\lambda_{t}+\varepsilon_{it}$$ (3)

$$GB_{it}=\alpha_{0}+\beta_{2}treated_{it}+\sum_{j=1}^{3}\gamma_{j}X_{it}+u_{i}+\lambda_{t}+\varepsilon_{it}$$ (4)

$$Resilience_{it}=\alpha_{0}+\beta_{3}treated_{it}+\delta GB_{it}+\sum_{j=1}^{3}\gamma_{j}X_{it}+u_{i}+\lambda_{t}+\varepsilon_{it}$$ (5)

#### 3.2.3. Moderating effect model.

To further test the moderating role of education, public environmental concern, and mineral resource dependence in the implementation of the policy, based on the moderation mechanism testing approach proposed by Baron and Kenny (1986) [52], this paper constructs a moderating effect model as follows:


$$\begin{array}{c} Resilience_{it}=\alpha_{0}+\beta_{1}pilot_{i}\times post_{t}\times Mod+\beta_{2}pilot_{i}\times post_{t}+\beta_{3}pilot_{i}\times Mod\\ +\beta_{4}post_{i}\times Mod+\sum_{j=1}^{3}\gamma_{j}X_{it}+u_{i}+\lambda_{t}+\varepsilon_{it} \end{array}$$ (6)

In the above model, pilot denotes the group dummy variable. It is assigned the value of 1 when it is a policy pilot, otherwise, it is assigned the value of 0. post denotes the time dummy variable, and it is assigned the value of 1 after the implementation of the policy, otherwise, it is assigned the value of 0. Mod denotes the moderating mechanism variables, which are education (Edu), public environmental concern (Pec), and mineral resources dependence (Mrd) in this study. $\beta_{1}$ is the estimated coefficient we are interested in, which indicates that the mechanism positively or negatively moderates the CACCP policy’s effect if it is statistically significant as a positive or negative value. Otherwise, it indicates that the mechanism does not play a moderating role.

### 3.3. Variable measurement and data source

#### 3.3.1. Outcome variable.

In this paper, Resilience represents the outcome variable of the CACCP policy, i.e., urban climate resilience. Based on this study’s conceptual definition of urban climate resilience, we constructed a system of indicators to measure it. With stabilization, coordination, and transformation capacity as the core elements, the system comprises 26 indicators, as shown in Table 1. In the indicator system, Stabilization reflects a city’s ability to defend and recover from climate disasters, using three dimensions: meteorological disaster warning, emergency response, and economic support [60,61]. Based on the fact that urban weather warning means and the number of people covered by them are the primary reflection of the early warning capacity and its scope of benefits; the adequacy of human resources and public health facilities to carry out rescue often determines the response capacity of a city when it encounters a climate disaster emergency; urban economic strength and financial strength of the government are the key forces to ensure that the city can restore stable operation quickly from a climate event. So, we have selected 11 representative indicators to reflect the stabilization capacity. Coordination reflects the ability of cities to reconcile economic growth with the response to climate change, using water security and carbon-neutral construction [20,62]. Reservoir construction, universal water supply, and sewage treatment levels are important measures to ensure urban production and life and to cope with climate change; increasing public green areas is conducive to attracting industrial investment and reducing carbon emissions. So, we have selected five representative indicators to reflect the coordination ability. Transformation reflects the evolutionary ability to reshape the architecture of urban systems and transform organizational functions [63–65]. The modernization level of urban infrastructure, scientific and technological progress, industrial structure upgrading, and decarbonization of the energy consumption structure are important bases and paths for cities to undergo organizational changes and adapt to future climate change. We have selected 10 representative indicators to reflect their transformation capacity.

**Table 1:** The indicator system for urban climate resilience measurement.

Target	Dimension 1	Dimension 2	Indicators
Urban climate resilience	Stability	Meteorological warning	Population coverage of television programs [15,16]
			Number of cell phone subscribers [15,16]
			Internet broadband access subscribers [53]
		Emergency response	Density of drainage pipes [17]
			Number of employees in public administration and social organizations [4,17]
			Urban road space per capita [5,8]
			Gas penetration rate [5,8]
			Number of beds in health facilities per 10,000 population [4]
			Health personnel per 10,000 population [4]
		Economic support	GDP per capita [19,54]
			Local general public budget expenditure [19,54]
	Coordination	Water security	Water supply penetration [55]
			Number of reservoirs [55]
			Sewage treatment rate [55]
		Carbon neutrality	Green space per capita in parks [56]
			Greening coverage in built-up areas [56]
	Transformation	Infrastructure	Length of drainage pipes in built-up areas [17–19]
			Investment in fixed assets for public infrastructure construction [17–19]
			Number of Internet broadband access ports [53]
			Length of long-distance fiber-optic cable lines [17–19]
		Technological innovation	Green patent applications [31,42]
			General public budget expenditure on science and technology as a share of GDP [15,31,42]
			Employment in scientific research and technical services [15,31,42]
		Structural adjustment	Industrial structure rationalization [57]
			Industrial structure advancement [58]
			Low-carbon energy consumption structure [59]

The raw data for the indicators are sourced from the China City Statistical Yearbook, the Yearbook of Meteorological Disasters in China, the China National Intellectual Property Administration database, and the National Bureau of Statistics database. The following indicators require special clarification: This paper adopts the measurement method proposed by Li and Wang (2019) for the low-carbon energy consumption structure indicator, which is expressed as the proportion of electricity and natural gas consumption in total energy consumption [59]. Following the method of Gan, Zheng, and Yu (2011), the indicator of industrial structure advancement is measured by the ratio of the tertiary industry’s output value to that of the secondary industry [58]. If this ratio is increasing, it indicates that the economy is upgrading towards a service-oriented direction. The indicator of industrial structure rationalization is measured using the inverse of the Theil index, as referenced by Yuan and Zhu (2018) [57], with the specific calculation formula as follows:


$$ISR_{i,t}=1/\sum {\mathrm{\backslash nolimits}}_{m=1}^{3}y_{i,m,t}\ln(y_{i,m,t}/l_{i,m,t})$$ (7)

In the formula, ISR denotes industrial structure rationalization, $y_{i,m,t}$ denotes the proportion of the added value of industry m in region i in the gross domestic product during period t, and $l_{i,m,t}$ denotes the proportion of employees in industry m in region i in the total employment during period t. The higher the ISR value, the more rational the industrial structure, and vice versa.

**Fig 1:**
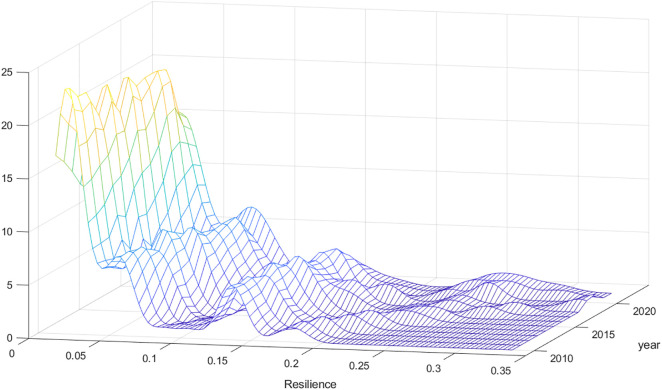
Kernel density distribution.

We used the entropy method (see S2 File) to measure the urban climate resilience of 57 cities in the Yellow River Basin from 2010 to 2022. The kernel density curve of Resilience was plotted as shown in Fig 1. It can be seen that the kernel density curves are all right-skewed, with peaks between 0 and 0.1. The center of the kernel density curve has shifted significantly to the right over time, indicating that although the urban climate resilience of most cities in the Yellow River basin remains low, it is showing a positive development trend of continuous improvement over time.

#### 3.3.2. Control variables.

We selected three control variables for analysis: the level of urbanization (urb), the number of days with extreme heat in a year (heatdays), and the number of days with a rainstorm in a year (rainstorms). The urb data were from the China City Statistical Yearbook. The raw data for heatdays and rainstorms were sourced from the National Centers for Environmental Information (NCEI) database, which is part of the National Oceanic and Atmospheric Administration (NOAA) in the United States. We counted the number of days with urban daily temperatures above 35°C and the number of days with rainfall exceeding 50 mm.

#### 3.3.3. Mediating and moderating variables.

In this study, the mediating mechanism variable (GB) data were from the CSMAR database. The moderating mechanism variables in this study are education (Edu), public environmental concern (Pec), and mineral resource dependence (Mrd). Pec was expressed using the urban residents' Baidu search index for “Environmental Pollution,” sourced from the Baidu Index website. Eduwas measured by the proportion of students enrolled in universities in the city to the number of household residents. Mrd was calculated using the share of urban employment accounted for by the number of people employed in the mining industry. The raw data for measuring Edu and Mrd were obtained from the China City Statistical Yearbook. All the data were standardized.

The data used in the analysis are attached as Supporting Information, as detailed in the S1 File. The descriptive statistics of the primary variable data are presented in Table 2.

**Table 2:** Data descriptive statistics.

Variables	Name	N	Mean	S.D.	Min	Max
Outcome variable	Resilience	741	0.0642	0.0587	0.0124	0.3345
Policy variable	treated	741	0.0729	0.2601	0.0000	1.0000
Control variables	heatdays	741	0.0841	0.1283	0.0000	1.0000
	rainstorms	741	0.0735	0.1502	0.0000	1.0000
	urb	741	0.5461	0.1556	0.1848	0.9595
Mediating variable	GB	741	0.2221	0.1869	0.0000	1.0000
Moderating variables	Edu	741	0.1436	0.2005	0.0000	1.0000
	Pec	741	0.1747	0.1904	0.0000	1.0000
	Mrd	741	0.1044	0.1365	0.0000	1.0000

## 4. Results

### 4.1. CACCP policy effect

**Fig 2:**
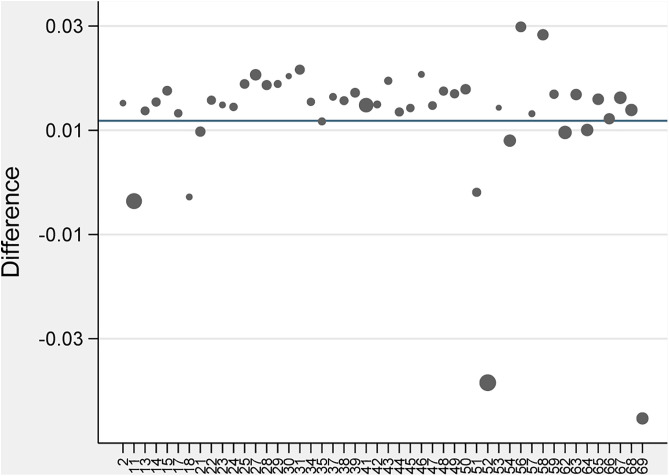
Empowerment of the control group.

**Fig 3:**
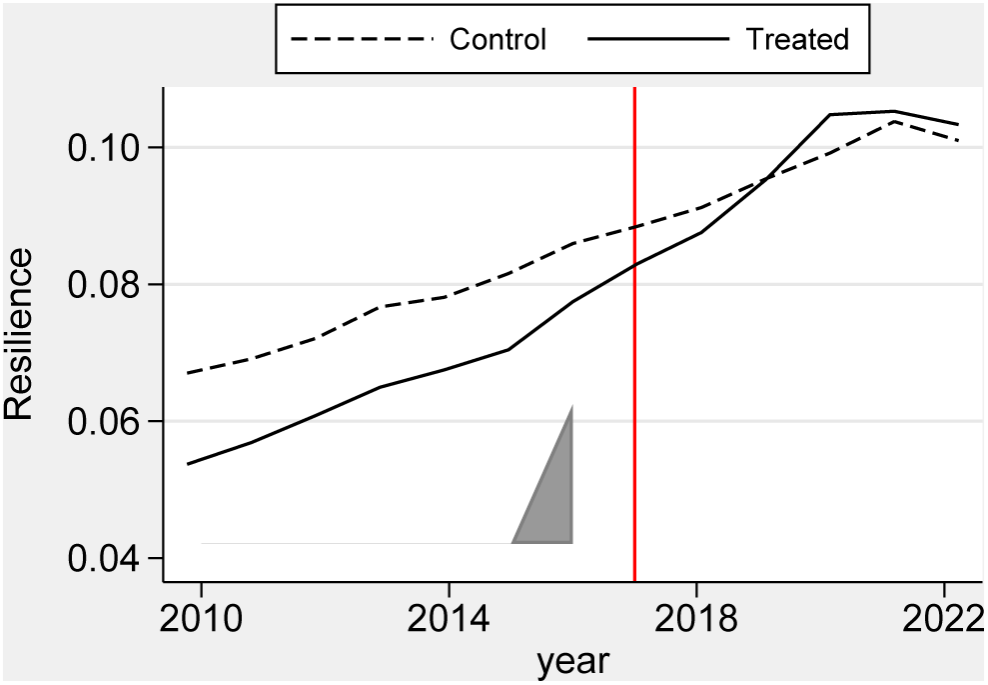
Dynamics of policy effects.

We employed the Synthetic DID method to evaluate the effect of the CACCP policy on urban climate resilience in the Yellow River Basin. Fig 2 depicts the results of the Synthetic DID method for assigning weights to individuals in the control group. Fig 3 depicts the temporal evolution of the policy’s impact. As illustrated in Fig 3, before the implementation of the CACCP policy (i.e., on the left side of the red vertical line), the Resilience of the experimental group was significantly lower than that of the control group. However, after the implementation of the policy (i.e., the right side of the red vertical line), the Resilience of the experimental group exhibited a notable acceleration. By 2019, it had surpassed that of the control group, indicating that the urban climate resilience of the pilot cities in the Yellow River Basin had benefited considerably from implementing the CACCP policy. As illustrated in Table 3, the mean treatment effect of the CACCP policy on the urban climate resilience in the Yellow River Basin is 0.0118, a statistically significant figure at the 5% level. This study confirms the validity of research hypothesis H1, further enhancing existing research conclusions on the effectiveness of climate adaptation policies [66,67]. Considering that the empirical data in this study were standardized to be between 0 and 1, the marginal contribution or average treatment effect value of the CACCP policy is only 0.0118. Therefore, the CACCP policy still requires further optimization to enhance its effectiveness.

**Table 3:** The average treatment effect of CACCP policy.

Resilience	ATT	Std. Err.	t	P > |t|	[95% Conf. Interval]	
treated	0.0118^**^	0.0048	2.48	0.013	0.0025	0.0212

Note: ** indicates a 5% statistical significance level.

### 4.2. Robustness tests

#### 4.2.1. Parallel trend test.

**Fig 4:**
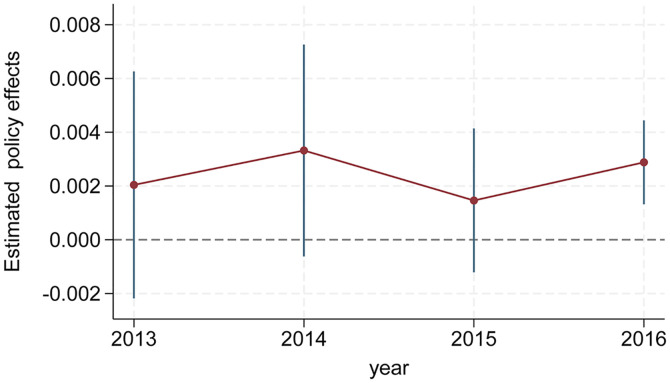
Parallel trend test.

While the Synthetic DID results do not rely heavily on the parallel trend assumption, this paper employs the event study method to conduct a parallel trend test, ensuring its validity. In order to ensure that there is a specific sample size before and after the pseudo-policy treatment, and in line with the Synthetic DID method on the characteristics of the control group of the spatiotemporal fit, we need to retain the sample size of a few years before the pseudo-policy treatment, so we choose 2013, 2014, 2015 and 2016 as the year of pseudo-policy implementation. We generated the pseudo-policy treatment variables for each year from 2013 to 2016. Then, we used the Synthetic DID method to carry out four estimations and got the pseudo-policy treatment estimation results, as shown in Fig 4. The results show that, before the policy implementation, from 2013 to 2016, the estimated policy effect was consistently close to 0, and except for 2016, the estimated policy effect was not statistically significant. The test results indicate that, prior to the implementation of the CACCP policy, there was no significant change in the gap between the experimental group and the synthetic control group in the outcome variable; that is, the parallel trends assumption was met, confirming the high validity of the Synthetic DID results in this study.

#### 4.2.2. Instrumental variable test.

We employed the instrumental variables method to address potential endogeneity issues in evaluating the CACCP policy. We selected the number of extreme rainfall days lagged by five years (L5_ERD) as the instrumental variable for this study, primarily for the following reasons: first, policymakers may prioritize cities with historically higher disaster risks (such as extreme rainfall) as pilot sites for climate adaptation initiatives; Policy makers may also tend to select cities with relatively better infrastructure, moderate risks, and greater potential for policy demonstration effects as pilot projects for climate adaptation construction. Therefore, L5_ERD meets the relevance condition (the instrumental variable may positively or negatively correlate with the policy). Second, the direct impact of historical disaster risks on current urban climate resilience has diminished over time and is now negligible. Therefore, L5_ERD satisfies the exogeneity condition. This study utilized the data on extreme rainfall days in Chinese cities published by Guo, Ji, and Zhang (2024) [68]. After standardizing the data from 2005 to 2017, an instrumental variable method was employed for estimation, with the results presented in Table 4. The results indicate that the instrumental variable L5_ERD passed the relevant tests, and the CACCP policy promoted climate resilience in the pilot cities. This result is consistent with the conclusions of this study, indicating that there are no serious endogeneity issues, and the results are robust.

**Table 4:** Results of the instrumental variable test.

Variable	treated	Resilience
treated		0.0789^*^ (0.0449)
*Constant*	0.2382^**^ (0.0946)	0.0508^***^ (0.0135)
*Controls*	YES	YES
City FE	YES	YES
Year FE	YES	YES
N	741	741
First-stage F-statistic	10.09^***^	
Hausman	26.15^**^	

Notes: Standard errors in parentheses; *** p < 0.01, ** p < 0.05, * p < 0.1;

Same as below.

#### 4.2.3. Mixed placebo test.

**Fig 5:**
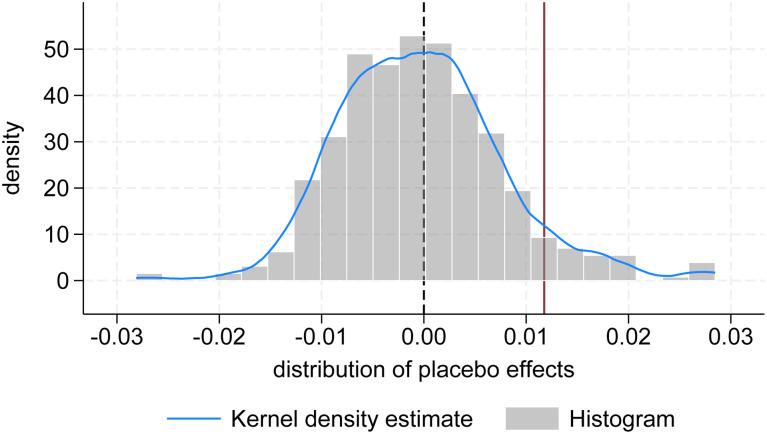
Mixed placebo test.

To enhance the credibility of the policy effect estimates in this study, we used a spatiotemporal mixed placebo method to test them. We simulated 500 random shocks and randomly generated pseudo-experimental groups and pseudo-policy treatment times, ultimately obtaining 500 estimates of the policy treatment. The kernel density curves of these estimates are shown in Fig 5. As illustrated in Fig 5, the estimates of policy effects derived from stochastic simulations exhibit a normal distribution. The coefficient estimates are predominantly distributed around 0, exhibiting a considerably smaller value than the estimates of the actual policy effect (the red vertical line indicates the estimate of the actual policy effect, 0.0118). Therefore, the test indicates that this study’s results are robust and not affected by other unobservable factors.

### 4.3. CACCP policy mechanisms test

#### 4.3.1. Mechanisms test results.

**Fig 6:**
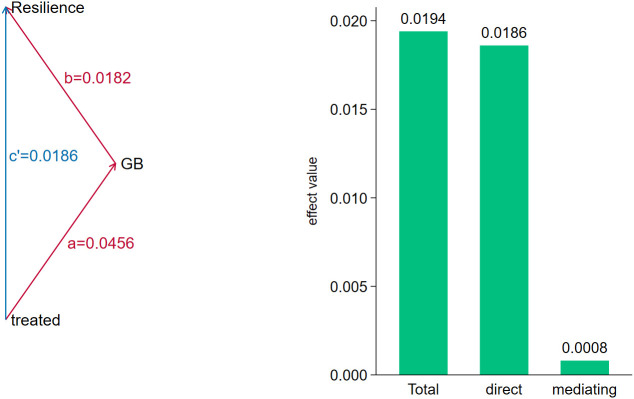
Transmission pathways and effect decomposition.

We further examined the policy’s mechanism for promoting urban climate resilience. Firstly, we tested the GB mediating variable based on the mediating mechanism model constructed in this paper. The test results are shown in Table 5. It is found that both the stepwise regression method and the Sobel test indicate that green bonds played a significant positive conduction role in the implementation of the CACCP policy. This study confirms the validity of research hypothesis H2, emphasizing the important role of financial instruments in climate adaptation [67,69, 70]. It also provides insights into the mechanisms behind EU financial integration [69] and private sector incentives [70]. At the same time, we drew the conduction process and effect decomposition diagram of this mediating mechanism (Fig 6), which shows that although the conduction effect of green bonds is relatively small at present (a×b=0.0008), it warns the Chinese government that it should strengthen the conduction role of the financial market for climate investment in the planning and action of climate adaptation policies.

**Table 5:** Results of the mediating mechanism test.

Variable	Resilience	GB	Resilience
treated	0.0194^***^ (0.0031)	0.0456^**^ (0.0192)	0.0186^***^ (0.0031)
GB			0.0182^***^ (0.0063)
*Constant*	0.0406^***^ (0.007)	0.3843^***^ (0.0431)	0.0336^***^ (0.0074)
*Controls*	YES	YES	YES
City FE	YES	YES	YES
Year FE	YES	YES	YES
N	741	741	741
Adj.R^2^	0.9306	0.7442	0.9314
Sobel Z	1.835^*^		

**Fig 7:**
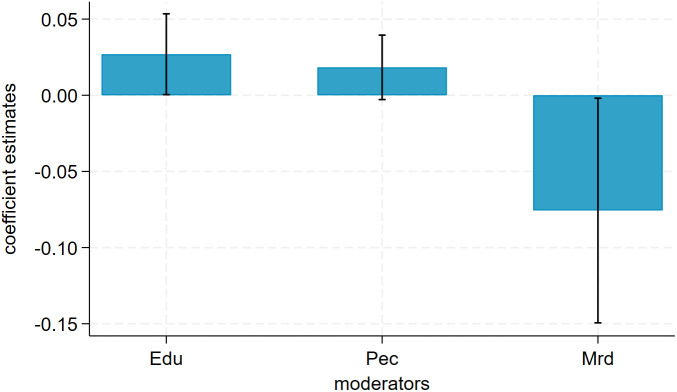
Moderating effect estimates and 95% confidence intervals.

Secondly, according to this paper’s moderating mechanism test model, we examined the moderating effects of education (Edu), public environmental concern (Pec), and mineral resource dependence (Mrd) on the CACCP policy. The results are shown in Table 6 and Fig 7. We can see that the estimates of the coefficient $\beta_{1}$ we are interested in are 0.0269, 0.0183, and −0.0757, respectively, and all are statistically significant. The findings indicate that the higher the level of urban education and public environmental concern, the more conducive it is to promoting the CACCP policy and enhancing its effectiveness. Consequently, research hypotheses H3 and H4 are validated. The findings also indicate that mineral resource dependence exerted a significant adverse moderating effect. In other words, the lower the urban mineral resource dependence, the higher the performance of the CACCP policy will be. Consequently, research hypothesis H5 is validated. We also found that, based on the use of uniformly standardized data treatment, the absolute magnitude of the moderating effect can be compared: Mrd>Edu>Pec. This study’s findings reveal the moderating role of urban human capital, cultural values, and economic structural characteristics in climate adaptation policies, and also provide quantitative evidence to complement research on equitable resilience [71] and racial vulnerability [72].

**Table 6:** Moderating mechanisms test results.

Variable	Resilience		
	(1)	(2)	(3)
pilot×postpilot×post	0.0039 (0.0042)	0.0024 (0.0038)	0.0209^***^ (0.0037)
pilot×post×Edu	0.0269^**^ (0.0135)		
pilot×Edu	-0.1932^***^ (0.0347)		
post×Edu	0.0464^***^ (0.0057)		
pilot×post×Pec		0.0183^*^ (0.0108)	
pilot×Pec		0.0088 (0.0165)	
post×Pec		0.0733^***^ (0.0065)	
pilot×post×Mrd			-0.0757^**^ (0.0376)
pilot×Mrd			0.1893^***^ (0.0348)
post×Mrd			-0.0288^***^ (0.0065)
Constant	0.0598^***^ (0.0075)	0.0604^***^ (0.0081)	0.0783^***^ (0.0088)
Controls	YES	YES	YES
City FE	YES	YES	YES
Year FE	YES	YES	YES
N	741	741	741
Adj.R^2^	0.9457	0.9492	0.9377

#### 4.3.2. Endogenous discussion.

On the one hand, we employed the instrumental variable method to address potential endogeneity issues in the mediating mechanism test. The CACCP policy instrument variable remains L5_ERD, with the rationale outlined in Section 4.2.2 of this paper. For green bonds (GB), the instrumental variable we selected is the 10-year lagged PM2.5 air pollution index, denoted as L10_Pollution. Historically, cities with severe air pollution may have more substantial incentives to issue green bonds. However, historical pollution levels (represented by the 10-year lagged PM2.5 index) are theoretically unlikely to directly influence current urban climate resilience. Therefore, L10_Pollution as the instrumental variable for GB satisfies the relevance and exogeneity conditions. The results of the instrumental variable method are presented in Table 7. The results indicate that, based on the passing of the F-statistic and Hausman tests, the effect of the treated on GB and the effect of GB on *Resilience* remain significantly positive, consistent with the results of the aforementioned mediation mechanism tests. Therefore, the mediation mechanism test results in this study are highly reliable.

**Table 7:** Endogeneity test results.

Variable	treated	GB	GB	Resilience
L5_ERD	-0.1755^**^ (0.0782)			
treated		0.8003^**^ (0.4038)		
L10_Pollution			0.2106^**^ (0.0908)	
GB				0.2717^**^ (0.1299)
*Constant*	0.2382^**^ (0.0946)	−0.1465 (0.1209)	−0.0057 (0.0485)	0.0612^***^ (0.0144)
*Controls*	YES	YES	YES	YES
City FE	YES	YES	YES	YES
Year FE	YES	YES	YES	YES
N	741	741	741	741
First-stage F-statistic	10.09^***^		16.14^***^	
Hausman	33.37^***^		106.69^***^	

**Fig 8:**
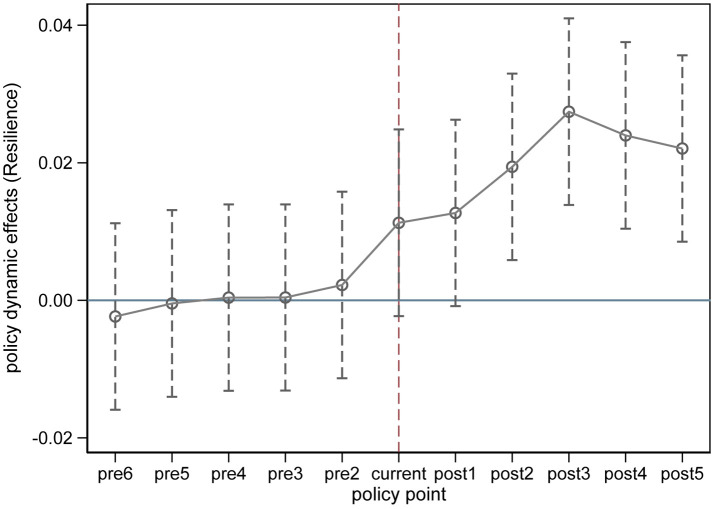
Parallel trend test results for DID method.

On the other hand, we employed the event study method to conduct a parallel trend test on the TWFE estimate of the CACCP policy effect, with the results shown in Fig 8. The findings indicate that the TWFE estimates of the policy effects satisfy the parallel trend assumption. This result suggests that the triple difference (DDD) estimation used in the moderation mechanism test possesses some methodological robustness. Additionally, the fit of the moderation effect model in this study is highly satisfactory (0.9457, 0.9492, and 0.9377). Based on the above analysis, the moderation mechanism test results exhibit a high reliability.

## 5. Discussion and conclusions

This paper focuses on China’s Climate-Adaptive City Construction Pilot Program (CACCP), utilizing panel data from 57 cities in the Yellow River Basin—a region characterized by ecological fragility—from 2010 to 2022. It employs Synthetic DID, stepwise regression, Sobel tests, and triple difference methods to empirically analyze the impact of the CACCP policy on urban climate resilience and its underlying mechanisms. The main research conclusions are: (1) Urban climate resilience is defined as a city’s comprehensive ability to adapt to long-term climate change trends, including stability, coordination, and transformation capabilities. Based on this, the study constructed an urban climate resilience assessment indicator system comprising 26 indicators. (2) Empirical results indicate that the CACCP policy significantly enhances urban climate resilience. This conclusion remains valid after a series of robustness tests. (3) Mechanism tests reveal that green bonds significantly mediate the CACCP policy’s enhancement of urban climate resilience; urban education and public environmental concern positively moderate the policy’s implementation effects, while mineral resource dependency exerts an adverse moderating effect.

The main similarities between this study’s findings and the latest research literature on climate adaptation are as follows: First, this study empirically confirms that the CACCP policy can significantly enhance urban climate resilience, consistent with the findings of Friesenecker et al. (2025) and Luo et al. (2025), which point to the positive impact of climate adaptation policies [66,67]. Second, this study’s definition of urban climate resilience aligns with the emphasis on the systemic and dynamic nature of climate resilience highlighted in the literature by Zorita et al. (2025) and Reckien et al. (2025), breaking away from a single-dimensional perspective [73,74]. Third, this study identified the mediating mechanism of green bonds, which, together with the research of Pitzén et al. (2025) and Osei et al. (2025), jointly emphasized the key role of financial instruments in climate adaptation [67,69,70]. This study’s main differences and innovations compared to existing literature are as follows: First, existing literature mainly focuses on climate adaptation policies in countries such as the European Union, the United States, and Australia [69,73,75,76]. This study focuses on ecologically fragile regions in China (the Yellow River Basin). It systematically examines the effects of China’s CACCP policy on enhancing urban climate resilience, providing valuable insights for developing countries. Second, this paper reveals the role of green bonds as a channel for implementing climate adaptation policies, providing insights into the institutional black box of EU financial integration [69] and private sector incentive mechanisms [70]. Third, the study finds that education and public environmental concerns can optimize policy effectiveness. At the same time, mineral resource dependence (the resource curse) has an inhibitory effect, providing quantitative evidence to complement research on equitable resilience [71] and racial vulnerability [72].

To further optimize the effectiveness of the CACCP policy, this paper proposes the following recommendations: First, innovate climate investment portfolios and strengthen the role of financial instruments. The government should promote the innovation of diversified climate finance tools such as climate bonds, climate credits, climate funds, and climate risk insurance to meet the financial needs of short-, medium-, and long-term climate adaptation construction projects. Second, increase investment in education and enhance educational equity. Continue to increase financial allocations to education, ensuring that the growth rate of education funding exceeds that of regular fiscal revenue; implement scholarship and grant systems to promote educational equity; incorporate courses on the relationship between humans and nature into education systems at all levels. Third, media publicity, community activities, and information disclosure should be utilized to raise public awareness of environmental issues. Use television, radio, newspapers, and social media platforms to disseminate environmental protection information; regularly organize activities such as Cleanup Day and Arbor Day to encourage residents to participate in practical actions to improve the environment; formulate uniform information disclosure standards, expand the scope of environmental information disclosure, increase the frequency of disclosure, and promote the construction of digital platforms. Fourth, actively promote green energy transition and reduce the city’s dependence on mineral resources. Establish special funds to support the development and utilization of non-fossil energy sources, such as wind and solar power; encourage enterprises to adopt advanced production technologies and equipment to improve energy efficiency; develop a circular economy model and strengthen international cooperation on clean energy technologies.

This paper also has some limitations. First, China’s climate change adaptation efforts started relatively late, and its statistical system is not yet fully developed. The design of the indicator system and data collection are also constrained, and further optimization of the indicator system design is needed. Second, while the Synthetic DID method used in this paper’s policy evaluation combines the advantages of the traditional DID and synthetic control methods, it also has methodological limitations, such as reliance on optimization algorithms for weight selection and difficulty handling multiple interventions. Third, the study focuses on China’s CACCP policy, which has specific regional and institutional limitations. In the future, we will continue to improve the urban climate resilience evaluation system and policy assessment methods. We also plan to expand existing research in the following areas: First, based on the dimension of social equity, we will explore the differentiated effects of CACCP policies in ethnic minority communities and low-income groups; second, we will strengthen cross-country comparisons to analyze the moderating role of institutional environments on policy outcomes; third, we will assess whether the CACCP policy has triggered systemic transformations, such as shifts toward a low-carbon industrial structure.

## Supporting information

S1 File Data. (XLS)

S2 File Entropy method. (DOCX)
