# Supplementary material for: How can climate adaptation policy enhance urban climate resilience? Empirical evidence from China
Source: PLoS One. 2025 Nov 6;20(11):e0335736. doi: 10.1371/journal.pone.0335736 (PMC12591477; doi:10.1371/journal.pone.0335736)
Supplement: S2 File — (DOCX) [file pone.0335736.s002.docx]

**The steps for the entropy method in this article are as follows:**

**Step 1:** Data standardization. The purpose of standardization is to homogenize dissimilar data. In this paper, we use formula (1) for standardization.

$\left\{\begin{matrix} X_{itj}^{'}=\frac{X_{itj}-X_{j\min}}{X_{j\max}-X_{j\min}}\text{，}\text{If X is a positive indicator} \\ X_{itj}^{'}=\frac{X_{j\max}-X_{itj}}{X_{j\max}-X_{j\min}}\text{，}\text{If X is a negative indicator} \\ X_{itj}^{'}=1-\frac{|X_{js}-X_{itj}|}{X_{j\max}-X_{j\min}}\text{，}\text{If X is a moderate indicator} \end{matrix}\right.$ (1)

In the formula, $X_{j}^{'}$ represents the data of the jth indicator in region i in year t.$X_{j\max}$ and $X_{j\min}$denote the maximum and minimum values of the jth indicator, respectively; $X_{js}$denotes the threshold value of the jth moderate indicator, which is set to 0 in this study based on the industrial structure rationalization measurement criteria proposed by Yuan and Zhu (2018)[63].

**Step 2:** Non-negative translation. Since the standardized data contains zero values and the subsequent steps require the standardized data to be logarithmized, a non-negative translation method is used to eliminate the influence of zero values. In this paper, a minimal number, 0.001, is added to all data.

**Step 3:** Calculate the information entropy of the indicator. The formulas are shown in (2) and (3) below. In the formula, $Y_{itj}$ is the proportion of the sample value. $S_{j}$is the information entropy of the jth indicator. M is the number of regions.

$Y_{itj}=X_{itj}^{''}/\sum_{t=1}^{T} \sum_{i}^{m} X_{itj}^{''}$(2)

$S_{j}=-k\sum_{t=1}^{T} \sum_{i}^{m} (Y_{itj}\ln(Y_{itj})),k=1/\ln(mT)$ (3)

**Step 4:** Calculate the utility value of the indicators. The utility value of the jth indicator is $E_{j}$, and the calculation formula is as follows.

$E_{j}=1-S_{j}$ (4)

**Step 5:** Calculate the weights of the indicators. The idea behind weight calculation is to use the value coefficient of the indicator information; the higher the value coefficient, the greater the importance for the evaluation. The calculation formula is as follows.

$W_{j}=E_{j}/\sum_{j}^{n} E_{j}$ (5)

**Step 6: Calculate the comprehensive score (*Resilience*). Finally, calculate the comprehensive score for each region and year based on the weight** $W_{j}$**of each indicator. The formula is as follows.**

$Resilience_{it}=\sum_{j}^{n} (W_{j}(X_{itj}^{''})^{T})$ (6)
